# Targeting oxidative stress and mitochondrial dysfunction via URG7 overexpression in an in vitro Parkinson’s disease neuronal model

**DOI:** 10.1038/s41598-026-38925-6

**Published:** 2026-02-19

**Authors:** Ilaria Nigro, Rocchina Miglionico, Ludovica Lela, Luigi Milella, Faustino Bisaccia, Maria Francesca Armentano

**Affiliations:** Department of Health Sciences, University of Basilicata, Via dell’Ateneo Lucano 10, Potenza, 85100 Italy

**Keywords:** Neurodegenerative diseases, Oxidative stress, Mitochondrial dysfunction, URG7 protein, 6-Hydroxydopamine (6-OHDA), Neuroprotection, Stress signalling, Mechanism of action, Molecular neuroscience, Cell death in the nervous system, Intracellular signalling peptides and proteins, Membrane proteins, Mitochondrial proteins, Membranes, Parkinson's disease, Molecular medicine, Mechanisms of disease

## Abstract

Neurodegenerative diseases represent a major healthcare challenge for aging populations, characterized by the progressive decline of neuronal function and subsequent brain atrophy. Despite differences in the brain regions affected and the underlying causes, these pathologies share common cellular and molecular mechanisms, including oxidative stress and mitochondrial dysfunction. Among neurodegenerative disorders, Parkinson’s disease (PD) is particularly associated with oxidative stress–driven dopaminergic neurodegeneration, in which reactive oxygen species (ROS) play a crucial role by contributing to cellular damage and neuronal death. This study investigates the role of Up-Regulated Gene 7 (URG7), a protein localized in the endoplasmic reticulum, in modulating oxidative stress in genetically engineered SH-SY5Y neuroblastoma cells overexpressing URG7, exposed to 6-hydroxydopamine (6-OHDA), a neurotoxin used as a Parkinson’s disease mimetic. We hypothesized that URG7 overexpression mechanistically modulates oxidative stress responses and mitochondrial integrity in neuronal cells subjected to Parkinson’s disease–related oxidative insult. Our results demonstrate that URG7 significantly reduces cell death and oxidative stress by enhancing cellular antioxidant defenses. Specifically, URG7 upregulates key antioxidant enzymes, including catalase and superoxide dismutase 2, and activates the Nrf2 signaling pathway, crucial for cellular detoxification. Furthermore, URG7 preserves mitochondrial membrane potential and regulates the expression of proteins involved in mitochondrial quality control, such as PINK1, Parkin, and DJ-1. Overall, these findings suggest that URG7 acts as a protective sentinel against oxidative stress and mitochondrial dysfunction, highlighting its potential as a therapeutic target for neurodegenerative diseases, particularly Parkinson’s disease. Further investigation into the molecular mechanisms of URG7, building on the biological evidence provided here, could pave the way for new neuroprotective strategies aimed at counteracting oxidative damage and promoting neuronal survival.

**Supplementary Information:**

The online version contains supplementary material available at 10.1038/s41598-026-38925-6.

## Introduction

Neurodegenerative diseases are among the leading causes of disability and morbidity worldwide and receive considerable attention due to their significant impact on aging societies^1^.

Among these, PD is the second most common neurodegenerative disease after Alzheimer’s disease and represents the fastest growing neurological disease in terms of prevalence, disability and mortality. PD is a progressive disorder characterized by the selective degeneration of dopaminergic neurons in the substantia nigra pars compacta and by the accumulation of misfolded α-synuclein in inclusions known as Lewy bodies. Clinically, PD manifests with cardinal motor symptoms, including resting tremor, rigidity, bradykinesia, and postural instability, together with a wide range of non-motor features such as cognitive impairment, sleep disturbances, and neuropsychiatric symptoms, which collectively have a profound impact on patients’ quality of life^2,3^. Although the precise mechanisms underlying dopaminergic neurodegeneration in PD remain incompletely understood, oxidative stress and mitochondrial dysfunction are widely recognized as central contributors to disease pathogenesis^4,5^. In dopaminergic neurons of the substantia nigra pars compacta, impairment of complex I of the mitochondrial respiratory chain results in increased ROS production, reduced ATP levels, depolarization of the mitochondrial membrane potential (ΔΨm), and activation of apoptotic pathways. Under physiological conditions, cells possess multiple antioxidant defense mechanisms composed of enzymatic systems capable of detecting ROS and counteracting oxidative damage, thereby maintaining redox homeostasis. A key role in the regulation of this homeostasis is played by the Nrf2–Keap1 signaling pathway, which coordinates the cellular antioxidant response, modulates inflammasome activity, and preserves mitochondrial integrity, thus contributing to the limitation of oxidative damage and neurodegeneration^6,7^. However, sustained oxidative stress disrupts this balance, and excessive ROS accumulation not only damages proteins, lipids, and nucleic acids but also promotes α-synuclein aggregation and Lewy body formation, contributing to the neurodegenerative process characteristic of PD^8–10^. In addition to direct neuronal damage, persistent oxidative stress promotes microglial activation and drives their polarization toward a pro-inflammatory phenotype, thereby amplifying neuroinflammatory responses that further exacerbate neuronal vulnerability^11^. Furthermore, mutations in genes strongly associated with familial forms of Parkinson’s disease, such as PINK1 and Parkin, which are fundamental for mitophagy, lead to the accumulation of damaged mitochondria and increased oxidative stress, while alterations in DJ-1 compromise cellular antioxidant defenses, resulting in increased ROS generation in PD^12–14^. Despite extensive research efforts, no curative treatment is currently available, and clinical management relies on symptomatic therapies, including pharmacological approaches such as levodopa and complementary non-pharmacological interventions^15,16^. Given the pivotal role of oxidative stress and mitochondrial dysfunction in PD pathogenesis, therapeutic strategies aimed at restoring redox balance and mitochondrial homeostasis represent a promising avenue to slow disease progression and mitigate neurodegenerative processes^17^.

Up-Regulated Gene clone 7 (URG7) is a 99 amino acid protein localized in the endoplasmic reticulum (ER), with an N-Lumen/C-Cytosol orientation^18,19^. Recently, our group characterized the activity of this protein in an in vitro neuronal model subjected to chemically induced ER stress, mimicking another hallmark of neurodegenerative diseases^20^, namely protein misfolding. URG7 has been shown to mitigate ER stress by modulating various markers of the unfolded protein response (UPR) to favor cell survival, promoting autophagy, and counteracting apoptotic activation. Moreover, URG7 influences the levels of misfolded proteins within the ER by enhancing ubiquitination and reducing protein aggregation. Finally, in this context a decrease in calcium release from the ER into the cytosol was observed, together with an unexpected overall reduction in intracellular ROS levels. This latter finding particularly attracted our interest, prompting us to further investigate the molecular mechanisms underlying this effect, also in the context of an in vitro model of neuronal oxidative stress. In the present study, therefore, we evaluated the activity of URG7 in neuroblastoma-derived cells exposed to oxidative stress induced by 6-hydroxydopamine (6-OHDA), a neurotoxic mimetic commonly used to model Parkinson’s disease. Although historically considered a synthetic neurotoxin, recent evidence indicates that 6-OHDA can be endogenously produced as an oxidative product of dopamine, with its levels detected in the brain and urine of Parkinson’s patients, suggesting a possible contribution to disease pathogenesis^21–23^.6-OHDA is selectively taken up by catecholaminergic neurons through the dopamine transporter (DAT), thereby reproducing the specific degeneration of dopaminergic neurons in the substantia nigra typical of Parkinson’s disease^24,25^. Once internalized, 6-OHDA generates reactive oxygen species and other toxic products that cause oxidative stress, mitochondrial dysfunction, and cell death via apoptosis or necrosis^23,26^. This model faithfully reproduces multiple pathological features of the disease, including the selective loss of dopaminergic neurons, mitochondrial damage, and activation of apoptotic pathways, making it a well-established experimental tool for investigating neurodegenerative mechanisms and testing neuroprotective strategies. Using this experimental model, we sought to investigate URG7’s ability to attenuate oxidative stress, hypothesizing its involvement in cellular detoxification mechanisms that protect against oxidative damage and promote cell survival. Specifically, this study evaluates, for the first time, the protective effect of URG7 against oxidative stress–induced mitochondrial damage.

Therefore, this work aims to determine whether URG7 can be considered a promising direct and/or indirect modulator of cellular stress–related pathways, paving the way for its potential therapeutic application.

## Results

### URG7 reduces cell death induced by 6-OHDA

**Fig. 1:**
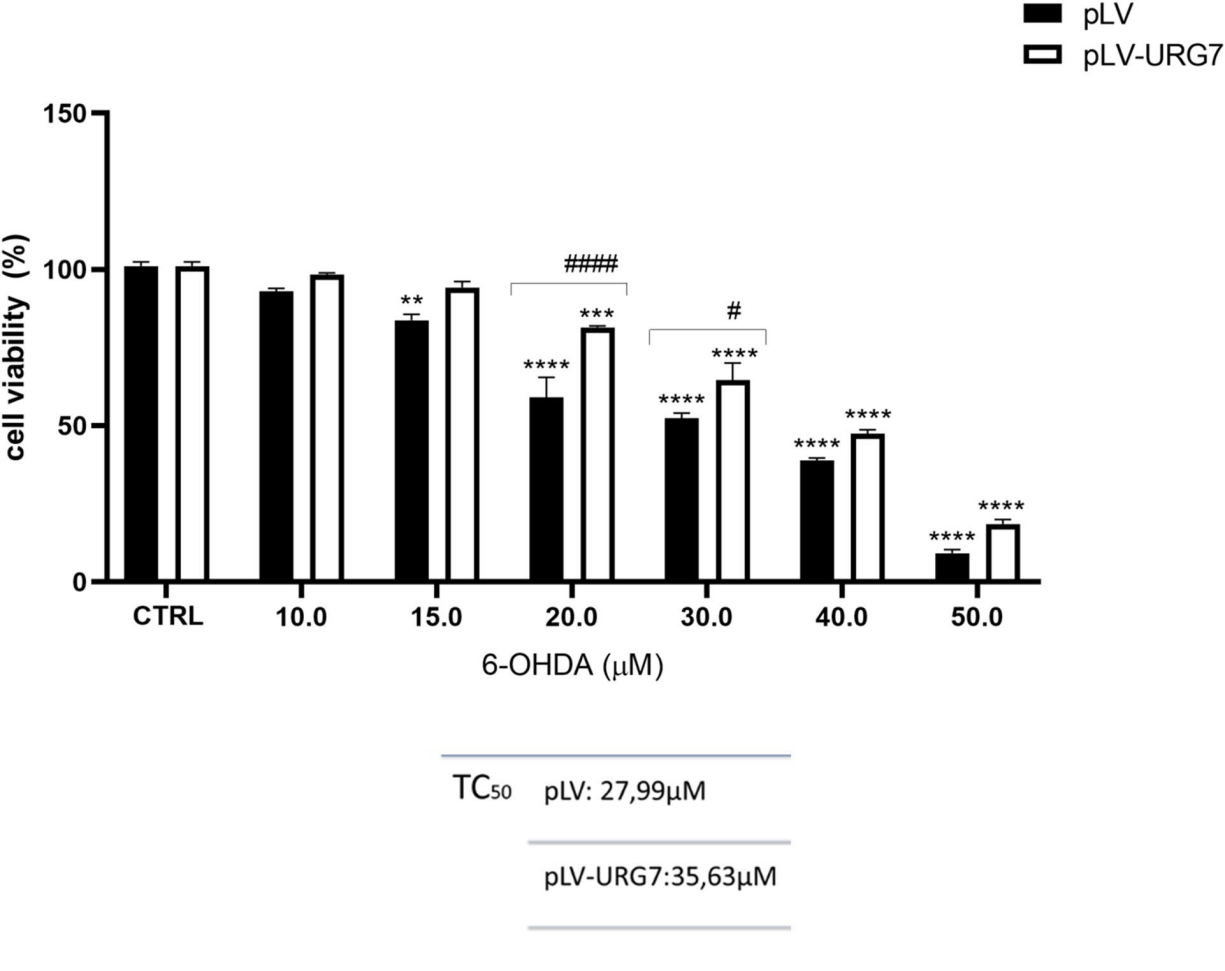
Cell viability assay and TC_50_ determination. Cells were exposed to the indicated concentrations of 6-OHDA for 24 h, and cell viability was assessed using the MTT assay, as described in the Materials and Methods section. The percentage of viable cells was calculated as the ratio of treated cells to untreated control cells (CTRL). Data are presented as mean ± standard error of the mean (SEM) from three independent experiments. Statistical significance was assessed using one-way ANOVA followed by Tukey’s post hoc test. Significance levels: # *p* < 0.05, **p < 0.01, ***p < 0.001, ****/#### *p* < 0.0001 [* comparison between untreated and 6-OHDA-treated cells; # comparison between 6-OHDA-treated pLV and pLV-URG7 cells].

To further investigate the potential neuroprotective role of URG7 in the context of neurodegenerative diseases, pLV-URG7 cells and control pLV cells were exposed to the neurotoxin 6-hydroxydopamine (6-OHDA), a Parkinsonian mimetic known to induce the formation of ROS, which in turn generate oxidative stress, mitochondrial dysfunction, and ultimately cell death—all hallmarks of Parkinson’s disease. As a preliminary step, the cytotoxic potential of 6-OHDA on neuronal cells was assessed using the MTT assay. Cells were treated for 24 h with different concentrations of 6-OHDA (50 µM, 40 µM, 30 µM, 20 µM, 15 µM and 10 µM). As shown in Fig. 1 and table, the two cell lines exhibited significantly different TC_50_ values, indicating a differential susceptibility to 6-OHDA-induced cytotoxicity associated with URG7 overexpression, and suggesting a protective role of URG7 under oxidative stress conditions.

**Fig. 2:**
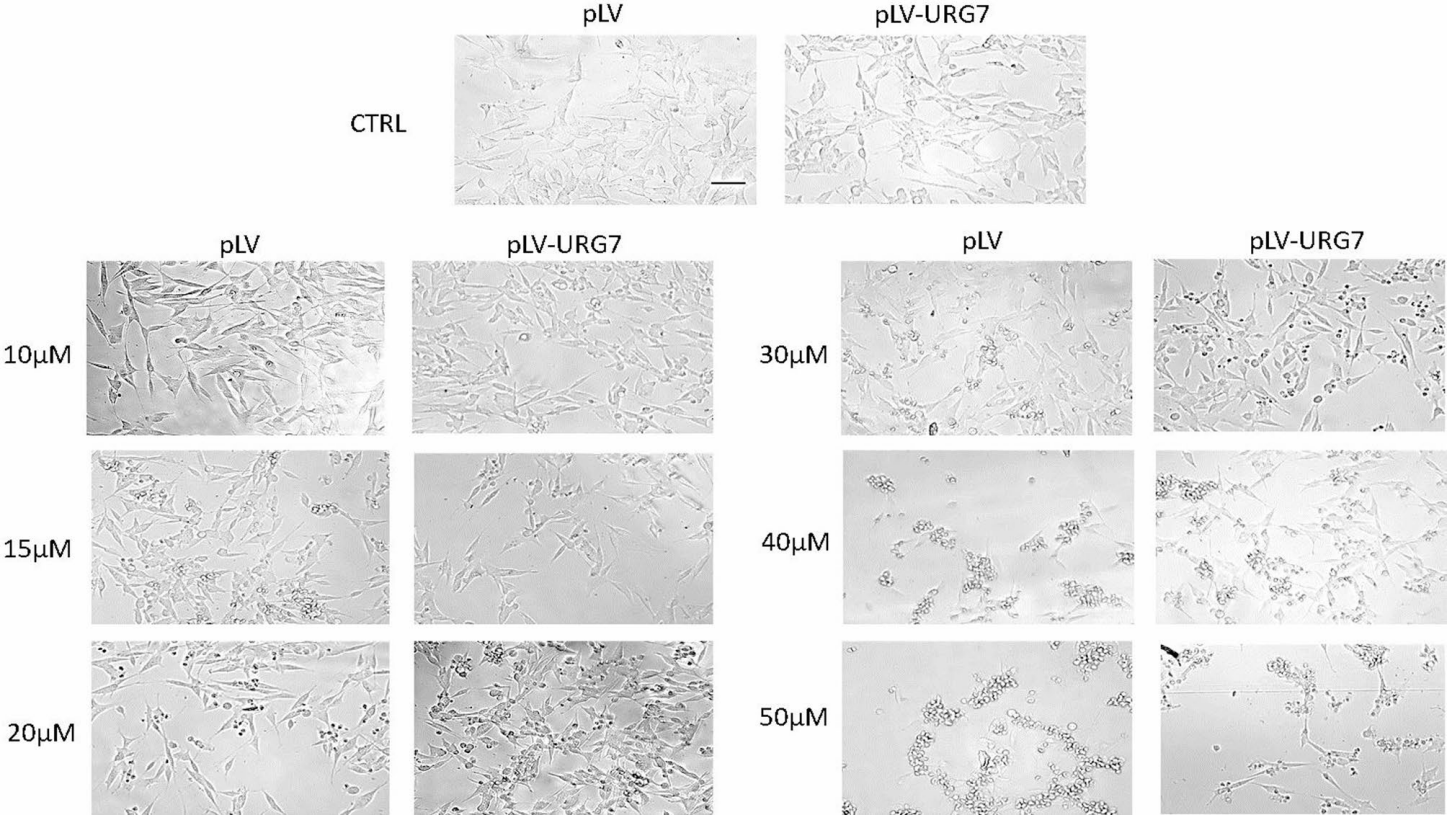
Morphological analysis of cells. Inverted phase contrast images showing changes in pLV and pLV-URG7 cells induced by different concentrations of 6-OHDA. Scale bars: 100 μm.

This difference in cytotoxicity was also evident through morphological analysis of the two cell lines treated with various concentrations of the neurotoxin, as visualized by light microscopy (Nikon Eclipse TS100) at 40X magnification (Fig. 2). In particular, starting from the concentration of 20 µM 6-OHDA, pLV cells showed clear morphological alterations compared to untreated controls and pLV-URG7 cells, including a marked reduction in cell number due to detachment, smaller cell bodies with a more rounded shape (rounding up), and retraction of neurite-like processes, consistent with increased neuronal damage under oxidative challenge.

### Protective effect of URG7 against intracellular ROS production induced by 6-OHDA neurotoxin

**Fig. 3:**
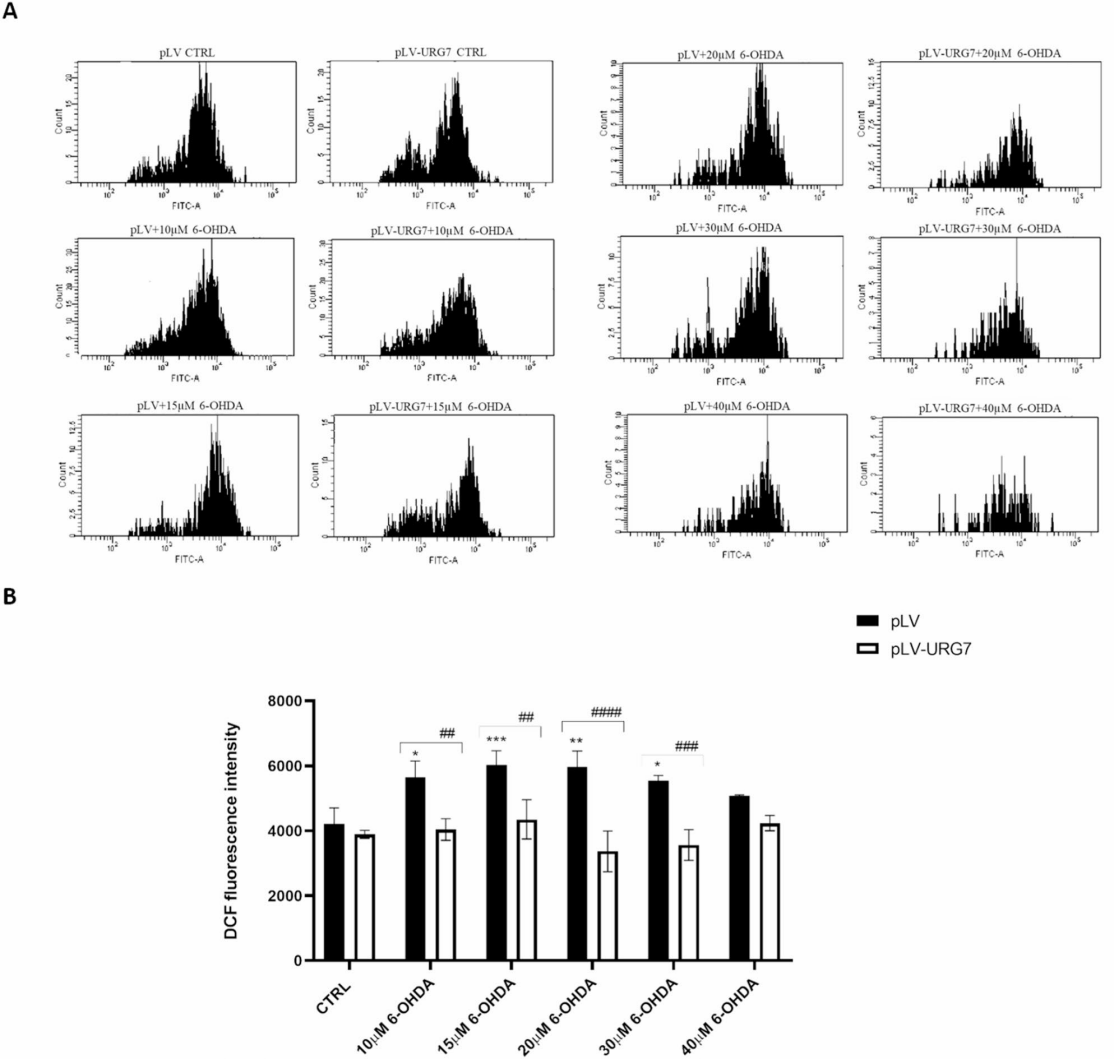
Quantification of intracellular reactive oxygen species (ROS). SH-SY5Y cells stably transfected with either pLV or pLV-URG7 were treated with various concentrations of 6-OHDA for 24 h. Intracellular ROS levels were measured using the fluorescent probe DCFH-DA and analyzed by FACScan flow cytometry. Representative fluorescence histograms (**A**) and quantitative analysis (**B**) are shown. Data are presented as mean ± standard error of the mean (SEM) from three independent experiments. Statistical significance was assessed using one-way ANOVA followed by Tukey’s post hoc test. Significance levels: *p < 0.05, **/##*p* < 0.01, ***/###*p* < 0.001, #### *p* < 0.0001 [* comparison between untreated and 6-OHDA-treated cells; # comparison between 6-OHDA-treated pLV and pLV-URG7 cells].

Intracellular ROS levels were assessed using the DCFH-DA fluorescent probe. SH-SY5Y pLV cells exposed to different concentrations of 6-OHDA (40 µM, 30 µM, 20 µM, 15 µM and 10 µM) for 24 h exhibited a significant increase in DCF signal compared to untreated control cells, reflecting a dose-dependent oxidative stress response. In contrast, this increase was not observed in SH-SY5Y pLV-URG7 cells, suggesting that URG7 expression effectively counteracts ROS generation (Fig. 3). Notably, a pronounced difference between the two cell lines was observed following treatment with 20 µM 6-OHDA, highlighting this concentration as a condition in which the ROS-modulating effect of URG7 is most evident, and therefore selected for subsequent experiments aimed at further investigating the underlying mechanisms.

### Effect of URG7 on markers involved in oxidative stress

Cells possess antioxidant defense systems comprising various enzymes that help maintain redox homeostasis and protect against oxidative damage. To explore the potential role of URG7 as a ROS scavenger, we assessed the expression levels of key antioxidant enzymes and transcription factors using RT-qPCR and Western blot analysis.

**Fig. 4:**
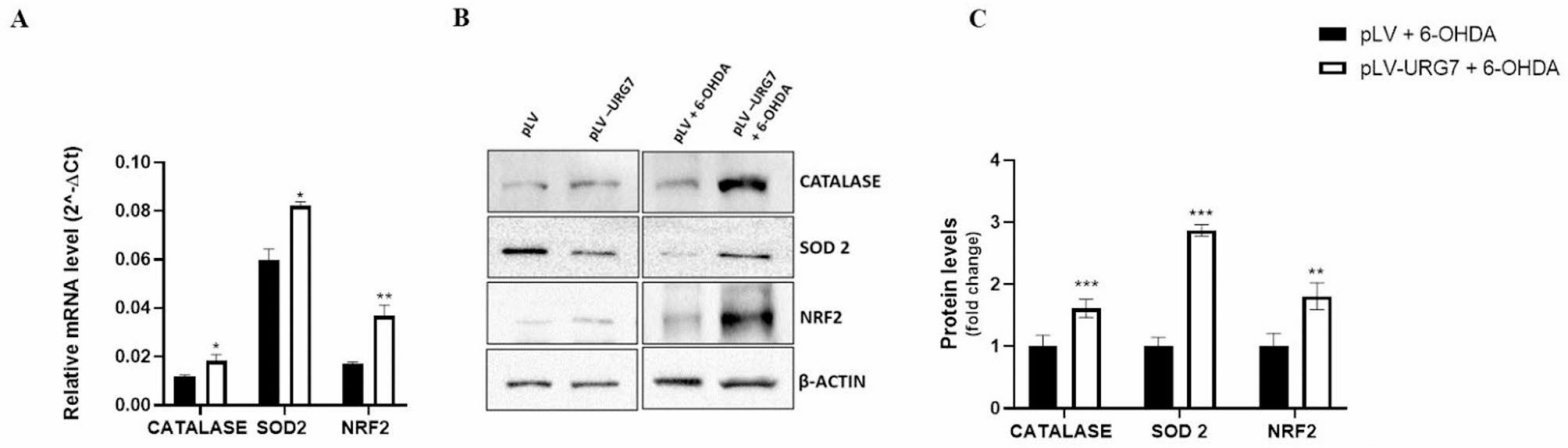
Effect of URG7 on markers involved in oxidative stress. (**A**) mRNA levels of Catalase, SOD2 and Nrf2 were quantified by real-time PCR in control cells (pLV) and in URG7-overexpressing cells (pLV-URG7) following treatment with 20 µM 6-OHDA for 24 h. β-actin was used as the internal control. Data are expressed as mean ± SEM (*n* = 3). Statistical analysis was performed using an unpaired, two-tailed t-test (GraphPad Prism 8.4.2); *p < 0.05, **p < 0.01. (**B**) Representative Western blotting images and (**C**) densitometric analysis of protein expression in SH-SY5Y cells stably transduced with either pLV vector or pLV-URG7, treated with 20 µM 6-OHDA for 24 h. β-actin was used as the loading control. Data are expressed as fold change relative to control cells treated with 6-OHDA (pLV + 6-OHDA), and shown as mean ± SEM from three independent experiments. Statistical significance was determined using an unpaired two-tailed t-test (GraphPad Prism 8.4.2). Significance levels: ** *p* < 0.01, *** *p* < 0.001.

As shown in Fig. 4A, pLV-URG7 cells exhibited increased transcript levels of Catalase, Superoxide Dismutase 2 (SOD2), and Nuclear factor erythroid 2–related factor 2 (Nrf2) compared to control (pLV) cells, suggesting that URG7 may contribute to cellular detoxification from ROS. These findings were corroborated by protein expression levels determined via Western blotting (Fig. 4B and C), supporting a coordinated regulation of antioxidant defenses.

**Fig. 5:**
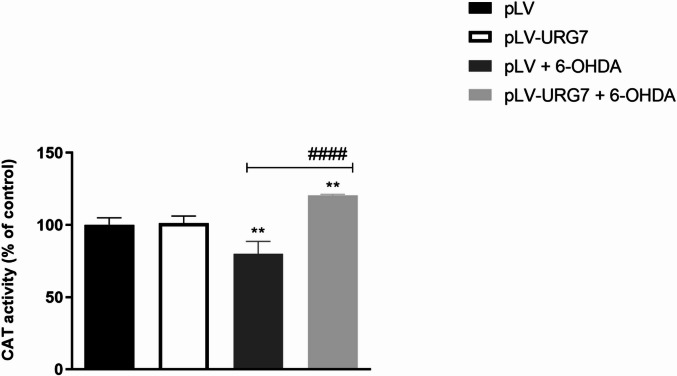
Effect of URG7 on catalase (CAT) activity in SH-SY5Y cells. Catalase activity was measured in pLV and pLV-URG7 cells following treatment with 20 µM 6-hydroxydopamine (6-OHDA) for 24 h. Data are presented as mean ± standard error of the mean (SEM) from three independent experiments. Statistical significance was assessed using one-way ANOVA followed by Tukey’s post hoc test. Significance levels: **p < 0.01, #### *p* < 0.0001 [* comparison between untreated and 6-OHDA-treated cells; # comparison between 6-OHDA-treated pLV and pLV-URG7 cells].

Catalase enzyme activity was assessed to further support gene and protein expression data. As shown in Fig. 5, treatment with 20 µM 6-OHDA for 24 h resulted in a significant ~ 30% reduction in catalase activity in pLV cells compared to untreated controls. In contrast, pLV-URG7 cells displayed increased catalase activity following oxidative challenge, suggesting preservation of enzymatic antioxidant function in the presence of URG7, which is consistent with the reduced intracellular ROS levels observed in these cells.

**Fig. 6:**
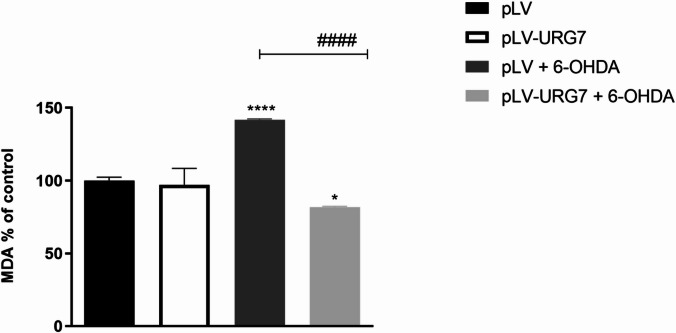
Effect of URG7 on MDA levels in SH-SY5Y cells. Malondialdehyde (MDA) levels were measured in pLV and pLV-URG7 cells following treatment with 20 µM 6-OHDA for 24 h. Data are presented as mean ± standard error of the mean (SEM) from three independent experiments. Statistical significance was evaluated using one-way ANOVA followed by Tukey’s post hoc test. Significance levels: ****/####*p* < 0.0001, *p < 0.05 [* Comparison between untreated and 6-OHDA-treated cells; #Comparison between 6-OHDA-treated pLV and pLV-URG7 cells].

Excessive ROS accumulation during oxidative stress is known to promote lipid peroxidation, leading to membrane damage and the formation of reactive byproducts such as malondialdehyde (MDA). Accordingly, pLV cells treated with 20 µM 6-OHDA for 24 h exhibited significantly elevated MDA levels compared to untreated controls (Fig. 6), indicating enhanced lipid peroxidation under oxidative conditions. Notably, URG7 overexpression markedly reduced MDA accumulation, consistent with a mitigation of oxidative membrane damage in URG7-expressing cells.

### Mitochondrial protective effect of URG7

**Fig. 7:**
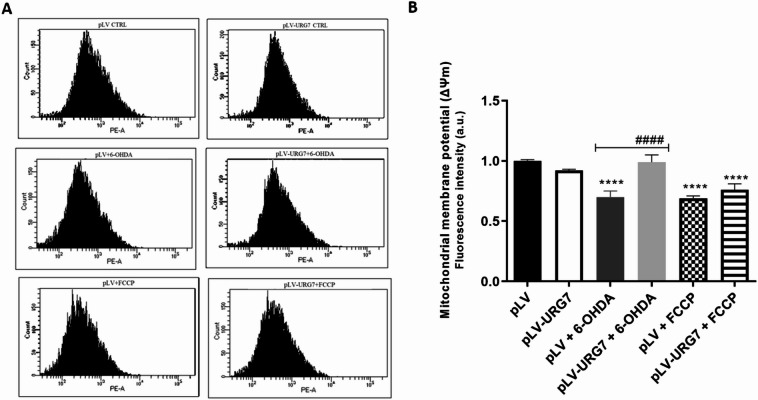
Effect of URG7 protein on mitochondrial membrane potential (ΔΨm). Control cells (pLV) and and URG7-overexpressing cells (pLV-URG7) were treated with 20 µM 6-OHDAfor 24 h and and mitochondrial membrane potential was assessed using MitoTracker Red CMXRos dye and flow cytometry. FCCP (5µM) was included as a positive control for mitochondrial membrane depolarization. Representative fluorescence histograms (**A**) and quantitative analysis (**B**) are shown. Data are presented as means ± Standard Error of the Mean (SEM) from three independent experiments. Statistical significance was calculated using one-way ANOVA followed by Tukey’s post hoc test. Significance levels: ****/####*p* < 0.0001 [*Comparison between untreated and 6-OHDA-treated cells; #Comparison between 6-OHDA-treated pLV and pLV-URG7 cells].

Mitochondria are a major source of reactive oxygen species and are themselves highly vulnerable to ROS-induced damage, which can lead to mitochondrial dysfunction. A key indicator of mitochondrial health is the mitochondrial membrane potential (ΔΨm), which reflects the organelle’s metabolic activity. Loss of ΔΨm disrupts mitochondrial function and cellular homeostasis, ultimately promoting apoptotic cell death. This process is a hallmark of neuronal degeneration. Given the results obtained, we investigated whether URG7 could preserve mitochondrial function under oxidative stress. Changes in ΔΨm were assessed by flow cytometry using the mitochondria-sensitive dye MitoTracker^TM^ Red CMXRos. As shown in Fig. 7, treatment of pLV cells with 20 µM 6-OHDA for 24 h led to an approximately 30% reduction in ΔΨm compared to untreated cells, indicating a marked impairment of mitochondrial function under oxidative stress. In contrast, pLV-URG7 cells maintained ΔΨm levels comparable to unstressed controls, demonstrating that URG7 confers a protective effect against 6-OHDA-induced mitochondrial damage.

**Fig. 8:**
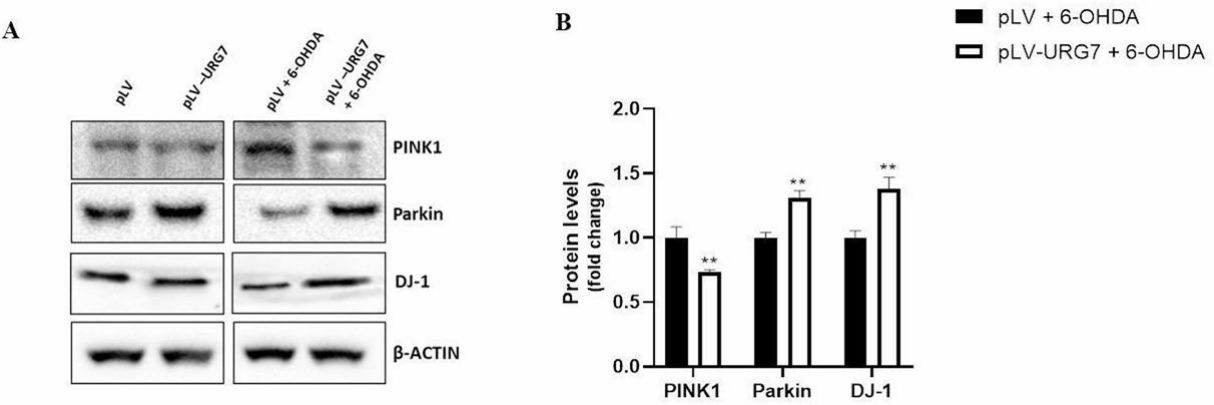
Effect of URG7 on the expression of proteins involved in Parkinson’s disease pathogenesis. (**A**) Representative Western blotting and (**B**) corresponding densitometric analysis of PINK1, Parkin, and DJ-1 expression in SH-SY5Y cells stably transduced with either the pLV control vector or the pLV-URG7 vector, following treatment with 20 µM 6-OHDA for 24 h. β-actin was used as the loading control. Data are expressed as fold change relative to control cells treated with 6-OHDA (pLV + 6-OHDA) and presented as mean ± standard error of the mean (SEM) from three independent experiments. Statistical significance was determined using an unpaired two-tailed t-test (GraphPad Prism 8.4.2). Significance levels: ** *p* < 0.01.

We also evaluated the expression of several Parkinson’s disease-related proteins involved in mitochondrial function, specifically PINK1, Parkin and DJ-1. Protein quantification results (Fig. 8) showed that, following 6-OHDA treatment, PINK1 expression was reduced in pLV-URG7 cells compared to control cells.

This finding suggests that mitochondrial membrane potential remains intact in URG7-expressing cells, as PINK1 is rapidly degraded under homeostatic conditions and accumulates on the outer mitochondrial membrane only when membrane potential is lost^27^. Parkin, a cytosolic E3 ubiquitin ligase, widely recognized for its neuroprotective role^28^, was found to be up-regulated in stressed pLV-URG7 cells compared to controls, indicating a potential enhancement of mitochondrial quality control mechanisms. Furthermore, the multifunctional protein DJ-1, known for its antioxidant properties and involvement in maintaining mitochondrial function and morphology^29^, was also found to be upregulated in the presence of URG7. Collectively, these data align with previous observations and support the conclusion that URG7 exerts a protective role against oxidative and mitochondrial stress.

## Discussion

Since the earliest studies on the pathogenesis of neurodegenerative diseases, it has become clear that these disorders share several common cellular phenotypes, including oxidative stress and mitochondrial dysfunction. It is now well established that oxidative stress, through the disruption of mitochondrial function and the induction of apoptosis, plays a central role in neuronal death and, consequently, in the development of neurodegenerative conditions such as Alzheimer’s disease, Parkinson’s disease, Amyotrophic Lateral Sclerosis, and Multiple Sclerosis^30–32^. A major challenge currently faced by researchers is the identification of novel therapeutic strategies capable of targeting and modulating the molecular and cellular mechanisms that drive the progression of these diseases.

We previously demonstrated that URG7, in a neuronal in vitro system subjected to proteotoxic stress, acts as a mitigator of endoplasmic reticulum (ER) stress. Specifically, it modulates markers of the unfolded protein response (UPR) in a way that favors cell survival, promotes protein ubiquitination to reduce aggregation, activates autophagy, and generally functions as an anti-apoptotic protein^20^. Despite these promising findings, proposing URG7 as a therapeutic target for Parkinson’s disease requires further investigation. To this end, we explored the activity of URG7 in the context of other dysregulated cellular processes associated with Parkinson’s disease. SH-SY5Y neuroblastoma cell lines—one stably overexpressing URG7 (pLV-URG7) and the corresponding control (pLV)—were treated with 6-hydroxydopamine (6-OHDA), a catecholaminergic neurotoxin widely used to establish experimental models of Parkinson’s disease. 6-OHDA is known to induce apoptosis in dopaminergic neurons, primarily through oxidative stress mechanisms^33,34^. It reacts rapidly and non-enzymatically with molecular oxygen, generating various ROS^35^. The proposed mechanism of 6-OHDA neurotoxicity involves ROS-mediated damage to cellular structures and metabolic systems^36–38^. Although the precise molecular pathways underlying 6-OHDA-induced neurotoxicity remain incompletely defined^39,40^ it is widely accepted that ROS generation and mitochondrial dysfunction are two central pathophysiological contributors to the neurodegenerative processes characteristic of Parkinson’s disease.

Therefore, in this study, we investigated whether URG7 could mitigate the cellular stress induced by 6-OHDA. In preliminary cell viability assays, we observed that cells overexpressing URG7 exhibited a significantly higher percentage of viable cells compared to control cells at the same 6-OHDA concentrations (Fig. 1). This observation was further supported by morphological analysis, which showed reduced cellular damage in URG7-expressing cells (Fig. 2). These initial findings provided essential operational insight for the design and execution of the subsequent experimental investigations. As previously discussed, redox imbalances are likely a major contributor to the cytotoxic effects of 6-OHDA in neuronal cells. This neurotoxin undergoes rapid oxidation in the presence of molecular oxygen, forming its corresponding p-quinone and a series of ROS, including the semiquinone radical, superoxide anion (O₂⁻), hydrogen peroxide (H₂O₂), hydroxyl radical (•OH), and hydroperoxyl radical (HO₂•)^38,41^. Given the established role of these reactive species in neurodegeneration, our first objective was to quantify ROS production and assess the ability of URG7 to promote cellular recovery following 6-OHDA-induced oxidative damage. Exposure to increasing concentrations of 6-OHDA for 24 h resulted in a dose-dependent rise in ROS levels in control (pLV) cells. In contrast, this increase was not observed in pLV-URG7 cells, indicating a protective, anti-stress role of URG7 (Fig. 3). To further explore the underlying mechanisms, we analyzed the expression and activity of key antioxidant enzymes, specifically superoxide dismutase (SOD) and catalase (CAT), using RT-PCR, Western blotting, and enzymatic activity assays. These enzymes constitute the primary endogenous defense against ROS and are known to mitigate 6-OHDA-induced cytotoxicity^42–45^.

We found that the expression of these antioxidant enzymes was increased in the pLV-URG7 cell line, a result that likely explains the reduced ROS levels observed following 6-OHDA-induced stress (Fig. 4). Specifically, we hypothesize that the protective effect of catalase (CAT) against 6-OHDA toxicity may be attributed to its enhanced ability to eliminate H₂O₂, which would in turn reduce the rate of 6-OHDA autoxidation and limit hydroxyl radical (•OH) generation via the Fenton reaction^33^. To support this, we measured the rate of H₂O₂ decomposition and found it to be significantly higher in pLV-URG7 cells (Fig. 5), confirming increased catalase enzymatic activity. As for superoxide dismutase (SOD), literature reports indicate that it reduces the formation of hydroperoxyl radicals (HO₂•), which are crucial in initiating lipid peroxidation^46–48^. Accordingly, we assessed the levels of malondialdehyde (MDA), a key byproduct of lipid peroxidation, and observed significantly lower MDA levels in pLV-URG7 cells compared to controls (Fig. 6), further validating the protective role of URG7. Overall, these results support the hypothesis that URG7 exerts an anti-stress function by enhancing cellular antioxidant defenses and reducing susceptibility to oxidative damage. The data indicate that URG7 does not act as a direct antioxidant but rather functions as an upstream regulator of cellular stress adaptation mechanisms, indirectly modulating redox homeostasis.

As previously noted, cells possess several defense mechanisms to counteract oxidative stress. Among these, the phase II antioxidant response is considered one of the most critical cellular defense pathways. This response is primarily regulated by the transcription factor Nrf2 (nuclear factor erythroid 2–related factor 2), which controls the constitutive and inducible expression of a wide array of cytoprotective genes.

Nrf2 targets include genes encoding for enzymes such as NAD(P)H quinone oxidoreductase-1 (NQO1), heme oxygenase-1 (HO-1), thioredoxins (Trx), glutathione S-transferase (GST), and its microsomal isoforms mGST1 and mGST2. It also regulates the expression of γ-glutamylcysteine synthetase (γ-GCS)—both its modifier subunit (GCLm) and catalytic subunit (GCLc)—as well as glutathione reductase (GR), superoxide dismutase-1 (SOD1), glutathione peroxidase (GPx), and numerous other phase I, II, and III detoxifying enzymes^49,50^.

Under basal conditions, Nrf2 is bound by the cytoplasmic repressor Keap1, which facilitates its interaction with an E3 ubiquitin ligase complex, leading to ubiquitination and subsequent proteasomal degradation of Nrf2^51,52^. Upon exposure to oxidative stress, Nrf2 is released from Keap1, translocates to the nucleus, forms heterodimers with other transcription factors, and binds to the antioxidant response element (ARE), thereby initiating the transcription of various antioxidant and cytoprotective genes^53,54^. Importantly, Nrf2 has been shown to play a critical role in mediating cellular responses to 6-OHDA-induced cytotoxicity^55^. To explore whether this pathway contributes to the reduced ROS levels observed in URG7-overexpressing cells, we evaluated the expression of Nrf2. As shown in Fig. 4, Nrf2 expression is significantly increased in pLV-URG7 cells following 6-OHDA treatment, indicating an association between URG7 overexpression and the activation of the Nrf2-mediated antioxidant response. In conclusion, the phase II antioxidant defense system represents a promising therapeutic target for neurodegenerative diseases, including Parkinson’s disease, and activation of these pathways is known to protect neurons from oxidative stress–induced cell death^56–59^. Overall, our results suggest that URG7 may modulate oxidative stress responses at a regulatory level, potentially involving transcriptional control mechanisms rather than acting exclusively through the activation of downstream antioxidant enzymes. These findings identify URG7 as a potential modulator of oxidative stress pathways and support further investigation of its therapeutic relevance in neurodegenerative conditions.

Mitochondria have long been a focal point of research due to their central role in human diseases, particularly neurodegenerative disorders. Evidence from pathology, toxicology, and genetics indicates that mitochondrial dysfunction is a key etiological factor in Parkinson’s disease^60,61^. Mitochondria are not only a major source of reactive oxygen species but are also highly vulnerable to oxidative damage. ROS can directly impair mitochondrial enzymes, induce mutations in mitochondrial DNA, and disrupt mitochondrial membrane permeability, as reflected by changes in membrane potential (ΔΨm).

In this study, treatment of pLV cells with 6-OHDA resulted in a marked loss of mitochondrial membrane potential, accompanied by elevated levels of ROS—a typical outcome of oxidative stress–induced mitochondrial dysfunction. Importantly, this mitochondrial impairment was significantly attenuated in URG7-overexpressing cells, as illustrated in Fig. 7, further highlighting the mitochondrial protective effect of URG7 under stress conditions. Several proteins have been implicated in the pathogenesis of Parkinson’s disease, including PTEN-induced putative kinase 1 (PINK1), DJ-1, and Parkin^62^. These proteins are either localized to the mitochondria or closely associated with mitochondrial processes, and as such, they play crucial roles in maintaining mitochondrial function and integrity. Their dysregulation has been strongly linked to the onset and progression of Parkinson’s disease, making them important molecular targets in the study of mitochondrial dysfunction in neurodegeneration.

PINK1 (PTEN-induced putative kinase 1) is a mitochondria-targeted serine/threonine kinase that plays a key role in mitochondrial quality control. Like many other mitochondrial proteins, the import of PINK1 into the mitochondria depends on the electrochemical gradient across the inner mitochondrial membrane, also referred to as the mitochondrial membrane potential (ΔΨm). Under normal conditions—when mitochondria are healthy and ΔΨm is maintained—PINK1 is efficiently imported into the inner mitochondrial membrane, where it undergoes proteolytic cleavage. The cleaved form is then released into the cytosol and rapidly degraded by the proteasome, preventing its accumulation^14,63^. This is precisely what we observed in pLV-URG7 cells exposed to 6-hydroxydopamine (6-OHDA): the expression level of PINK1 in these cells remained comparable to that of unstressed control cells (Fig. 8), indicating the preservation of mitochondrial integrity. Notably, PINK1 levels in stressed pLV-URG7 cells were significantly lower than those in stressed pLV control cells, further supporting the protective effect of URG7 on mitochondrial function. It is well established that under conditions of mitochondrial damage or loss of membrane potential, PINK1 import into the mitochondria is impaired. As a result, PINK1 accumulates on the outer mitochondrial membrane, serving as a signal for the recruitment of Parkin and the initiation of mitophagy in response to mitochondrial dysfunction. The absence of PINK1 accumulation in URG7-expressing cells suggests that URG7 helps maintain mitochondrial health, even under oxidative stress conditions^63–65^.

At this point in the pathway, PINK1 normally recruits Parkin, a cytosolic E3 ubiquitin ligase, to the outer mitochondrial membrane. Once localized to the mitochondria, Parkin is phosphorylated and activated by PINK1, initiating the selective degradation of damaged mitochondria via mitophagy^27^. In our study, this mitophagic mechanism does not appear to be activated in pLV-URG7 cells, as PINK1 does not accumulate on the outer mitochondrial membrane—consistent with the presence of functionally intact mitochondria. Interestingly, this pathway is also not effectively activated in the pLV control cells, despite the presence of mitochondrial damage. Analysis revealed low expression levels of Parkin in these cells, suggesting that dysfunctional mitochondria are not being targeted for degradation, ultimately leading to apoptosis. Moreover, literature reports indicate that Parkin is not only involved in mitophagy, but is also associated with the maintenance of healthy mitochondrial function. Loss of Parkin activity has been linked to increased oxidative stress and impaired function of mitochondrial complex I^66,67^, further supporting our findings and underscoring the relevance of Parkin expression in cellular resilience to oxidative damage. Moreover, the higher expression of Parkin observed in URG7-overexpressing cells could contribute to mitochondrial homeostasis through mechanisms independent of mitophagy, such as supporting respiratory chain stability and reducing ROS production, as previously reported^27,68^. Since recent studies have shown that PINK1 and Parkin localize at ER–mitochondria contact sites, where they not only modulate inter-organellar crosstalk, but also regulate key processes such as Ca²⁺ homeostasis, mitochondrial quality control, and the execution of mitophagy-hallmarks profoundly altered in major neurodegenerative diseases^69,70^-it is plausible to hypothesize that URG7, given its known cytoprotective functions and its localization at the endoplasmic reticulum, may likewise contribute to the structural and functional regulation of MAMs, thereby influencing the communication between the ER and mitochondria.

Another protein evaluated in this study is DJ-1, which is expressed in both the brain and peripheral tissues, with a predominantly cytosolic subcellular distribution^71–73^. DJ-1 is a multifunctional protein with several roles relevant to neuroprotection^74^. It has been suggested to exert its protective effects primarily through antioxidant activity and it is known to influence mitochondrial function and morphology^75^ as well as to activate autophagy pathways^76^. DJ-1 appears to exert its cytoprotective functions by acting as both a sensor and a scavenger of ROS under oxidative stress conditions. This antioxidant capacity is largely attributed to its cysteine residues, particularly Cys106, which undergo oxidation in a way that detoxifies ROS and limits cellular damage^29,77^. Beyond its antioxidant properties, DJ-1 has also been implicated in transcriptional regulation. It has been shown to function as a co-activator of transcription factors involved in cellular defense mechanisms, particularly those responsible for protecting cells from oxidative stress-induced apoptosis^78–80^. In this study, we observed that DJ-1 expression was significantly upregulated in pLV-URG7 cells following treatment with 6-OHDA, compared to pLV control cells (Fig. 8). This finding further supports the hypothesis that URG7 confers cellular protection by enhancing antioxidant defenses and modulating stress-responsive pathways.

Overall, the results obtained following exposure to the oxidative stressor 6-OHDA corroborate and extend previous findings, confirming that URG7 plays an active role in cellular detoxification mechanisms and promotes cell survival under stress conditions. In the present study, we demonstrated that URG7 mitigates oxidative damage by activating endogenous antioxidant defense systems, preserving mitochondrial membrane potential, and modulating the expression of key mitochondrial proteins implicated in the pathogenesis of Parkinson’s disease, thereby contributing to overall cellular protection.

In conclusion, the results of this study indicate that URG7 may act as a “sentinel” protein, intervening at an early stage of the cellular stress response and contributing to the coordination of molecular pathways involved in the maintenance of homeostasis. By providing the first evidence of URG7 involvement in the regulation of oxidative stress and the preservation of mitochondrial integrity in a Parkinson’s disease–related cellular model, this work identifies URG7 as a previously unexplored regulator of neuronal stress-response mechanisms.

Overall, the data highlight the biological relevance and therapeutic potential of URG7 in the context of Parkinson’s disease, supporting its further investigation as a possible target for neuroprotective strategies aimed at mitochondrial dysfunction and oxidative stress. Beyond its antioxidant and mitochondrial protective roles, the results suggest that URG7 contributes to the maintenance of cellular homeostasis under neurotoxic stress conditions, coordinating adaptive responses such as protein quality control, redox balance, and mitochondrial function.

Future studies will be required to validate these effects in in vivo models of Parkinson’s disease and to clarify the molecular interactions of URG7 within neuronal stress-response pathways. Taken together, these findings provide new insights into the role of URG7 in neuronal protection and open the way to the development of innovative therapeutic strategies for Parkinson’s disease and, potentially, for other neurodegenerative disorders characterized by oxidative stress and mitochondrial dysfunction.

## Materials and methods

### Reagents and antibodies

Dulbecco’s Modified Eagle’s Medium/Nutrient Mixture F-12 (1:1) (DMEM/F12-HEPES) medium was purchased from Corning ((#10090-CV Corning, NY, USA). Dulbecco’s Phosphate-Buffered Saline solution (DPBS #ECB4004L), penicillin–streptomycin solution (#ECB3001) and Fetal Bovine Serum (FBS #ECS5000L) were obtained from EuroClone (Milan, Italy). Dimethyl sulfoxide (DMSO #D8418), Trypsin–EDTA Solution (#T4049), Bovine Serum Albumin (#05470), 2′,7′-Dichlorofluorescin diacetate (#D6883), Bradford Reagent (#B6916), Thiazolyl Blue Tetrazolium Bromide (MTT, #475989), 6-Hydroxydopamine hydrochloride (#H4381), 2-thiobarbituric acid (#T5500), trichloroacetic acid (#T6399), Carbonyl cyanide p-(trifluoromethoxy)phenylhydrazone (FCCP) (#C2920) were purchased from Sigma Aldrich-Merck (Saint Louis, MO, USA). RIPA buffer (#9806) and Protease Inhibitor Cocktail (#5871) were purchased from Cell Signaling Technology (CST, Danvers, MA, USA). Quick-RNA™ MiniPrep kit was purchased from Zymo Research (#R1054 Irvine, CA, USA)., Pluronic F-127 (#10767854) and MitoTracker™ Red CMXRos Dye (#10767854) were purchased from Thermo Fisher Scientific (Waltham, MA, USA). High Capacity cDNA Reverse Transcription kit was purchased from Applied Biosystem (#4374967 Foster City, CA, USA). iTaqTM Universal SYBR Green Supermix was purchased from BIO-RAD (#1725121 Hercules, CA, USA). Oligonucleotides were purchased from Eurofins Genomics (Ebersberg, Germany). Primary antibodies specific for β-actin (#3700), anti-mouse IgG-HRP-linked (#7076), anti-rabbit IgG-HRP-linked (#7074), PINK1 (#6946), DJ-1 (#5933) and Catalase (#12980) were purchased from Cell Signaling Technology (CST, Danvers, MA, USA). Primary antibodies specific for SOD2 (#66474-1-Ig) and Nrf2 (#16396-1-Ig) were obtained from Proteintech. Antibodies anti-Parkin (#702785) was obtained from Invitrogen.

### Cell culture and treatments

The immortalized neuroblastoma cell line SH-SY5Y was purchased from the Cell Factory-ICLC (Genova, Italy; cell line catalog code: HTL95013). The SH-SY5Y cell line was selected as a well-established in vitro neuronal model widely used to investigate oxidative stress and mitochondrial dysfunction in Parkinson’s disease–related experimental contexts^81^. This cell line was engineered to stably overexpress the URG7 protein (obtained via lentiviral infection, as previously described^20^, and henceforth referred to as pLV-URG7). A corresponding control cell line (referred to as pLV) was also generated and used in all subsequent experiments. The cells were maintained in DMEM/F-12-HEPES medium supplemented with 10% FBS, 100 U/mL penicillin, and 100 µg/mL streptomycin, at 37 °C in a 5% CO₂ atmosphere, and were passaged every 48 h. All experiments were performed using cells at different passages, always below passage 15. To induce oxidative stress, cells were initially treated with different concentrations of 6-OHDA (50 µM, 40 µM, 30 µM, 20 µM, 15 µM, and 10 µM) for 24 h. Once the 50% toxic concentration (TC_50_) was determined, a final concentration of 20 µM 6-OHDA was selected for subsequent experiments. The TC₅₀ was calculated using GraphPad Prism software (GraphPad, La Jolla, CA, USA). The 6-OHDA powder was dissolved in H₂O to prepare a stock solution at a final concentration of 50 mM.

### Measurement of cell viability and observation of morphological changes

The cytotoxicity of 6-OHDA on cell lines was evaluated using the MTT [3- (4,5 -dimethylthiazol-2-yl)-2,5-diphenyltetrazolium bromide] colorimetric assay. pLV-URG7 and pLV cells were seeded into 96-well plates (2.5 × 10^4^ cells per well) and incubated for 24 h. The cells were then treated with different concentrations of the neurotoxin (50 µM, 40 µM, 30 µM, 20 µM, 15 µM and 10 µM) for an additional 24 h. After treatment, the media were removed and the cells were incubated with 100 µL of 0.75 mg/mL MTT solution for 4 hours in the dark at 37 °C. Formazan crystals formed by metabolically active cells were then solubilized with 100 µL of a 1:1 DMSO: isopropanol mixture per well. Absorbance was measured at 570 nm with background subtraction at 630 nm using a Multiskan Go spectrophotometer (Thermo Scientific, Waltham, MA, USA). In parallel, both cell lines were also seeded into 12-well plates (1.5 × 10^5^ cells per well) and incubated for 24 h. They were then treated with the same range of 6-OHDA concentrations for another 24 h, and cell morphology was assessed using phase-contrast microscopy (Nikon Eclipse).

### Determination of intracellular reactive oxygen species (ROS)

To measure the intracellular ROS levels, 1 × 10^5^ SH-SY5Y cells stably expressing URG7 and mock transduced control cells were seeded into 24-well plates and exposed to 6-OHDA at concentrations of 40 µM, 30 µM, 20 µM, 15 µM and 10 µM for 24 h. Following treatment, the cells were incubated with 1 µM 2,7-dichlorodihydrofluorescein diacetate (DCFH-DA) for 30 min at 37 °C in a humidified atmosphere containing 5% CO_2_. After incubation, the cells were harvested by trypsinization, resuspended in PBS and analyzed using a FACSCanto II flow cytometer (BD Biosciences), with excitation at 488 nm and emission detected at 530 nm. Fluorescence values were normalized to the number of viable cells assessed by flow cytometry (FSC/SSC event counting).

### RNA extraction and cDNA synthesis

pLV-URG7 and pLV cells were seeded into 12-well plates (1.5 × 10^5^ cells per well) and, the following day, were treated with 20 µM 6-OHDA for 24 h. At the end of the treatment, the cells were pelleted and RNA extraction was performed using Quick-RNA^TM^ MiniPrep kit (Zymo Research; Irvine, CA), with purification achieved through multiple centrifugation steps using filter columns, according to manufacturer’s protocol. RNA concentration and purity were assessed spectrophotometrically by measuring absorbance at 260 nm using a Multiskan^TM^GO Microplate Spectrophotometer (Thermo Fisher Scientific). The absorbance ratios at 260/280 and 260/230 nm were used as indicators of contamination by proteins and phenols, respectively. cDNA synthesis was performed using 1 µg of total RNA as a template with the High-Capacity cDNA Reverse Transcription Kit (Applied Biosystems; Waltham, MA), on a peqSTAR 96 Universal Thermo Cycler (EuroClone; Siziano, PV, Italy), following the program recommended by the kit manufacturer.

### Real-time PCR

Amplification and relative quantification of cDNA templates was performed by Real Time PCR using a 7500 Fast Real-Time PCR System (Applied Biosystems) and iTaq-Universal-SYBR^®^ Green Supermix (Bio-Rad; Hercules, CA). To verify PCR specificity, melting curve analysis was conducted on all amplified products. RT-PCR results are expressed using the 2^−ΔCt^ method, with β-actin used as the endogenous reference gene. Primers were designed, using the AlleleID program, to span exon–exon junctions, in order to prevent amplification of genomic DNA (see Table 1).

**Table 1:** Primers used in reverse transcription-quantitative polymerase chain reaction assay.

Gene	Accession number	Forward primer	Reverse primer
β-actin	NM_001101.3	5’CCT GGC ACC CAG CAC AAT-3’	5’-GCC GAT CCA CAC GGA GTA CT-3’
SOD 2	NM_000636.4	5’-CCG ACC TGC CCT ACG ACT AC- 3’	5’-AAC GCC TCC TGG TAC TTC TCC- 3’
CATALASE	NM_001752.4	5’-ATA CCT GTG AAC TGT CCC TAC CG-3’	5’-GTT GAA TCT CCG CAC TTC TCC AG-3’
Nrf2	NM_001145412.3	5’-AAC TAC TCC CAG GTT GCC CA-3’	5’-CAT TGT CAT CTA CAA ACG GGA A-3’

### Catalase activity

Catalase (CAT) activity was measured as previously described^82^, with minor modifications. Briefly, 5 µL of cell lysate from pLV-URG7 and pLV cells, treated or untreated with 20 µM of 6-OHDA, was mixed with 895 µL of potassium phosphate buffer (0.5 M, pH 7.2) and 100 µL of 400 mM H_2_O_2_. The rate of enzymatic decomposition of H_2_O_2_ was monitored spectrophotometrically at 240 nm using a SPECTROstar Nano reader (BMG Labtech, Ortenberg, Germany). CAT activity was calculated using the molar extinction coefficient of H_2_O_2_ (43.6 mM^− 1^ cm^− 1^) and expressed as percentage relative to the untreated control group (considered as 100%).

### TBARS assay

Lipid peroxidation was assessed using TBARS (Thiobarbituric Acid Reactive Substances) assay. One of the major end products of lipid peroxidation, malondialdehyde (MDA), was quantified by a colorimetric reaction with 2-thiobarbituric acid (TBA). Cell culture medium from cells stably overexpressing URG7 and the corresponding control cells - treated or untreated with 20 µM 6-OHDA, was mixed with trichloroacetic acid (TCA, 10%) and TBA (0.67%), then incubated at 95 °C for 15 min. After cooling to room temperature, absorbance was measured at 532 nm.

### Western blotting analysis

Identification and relative quantification of cellular proteins was performed by Western blot analysis. For protein analysis of both primary and immortalized cells, cell pellets were collected after seeding in 6-well plates at a density of 2.5 × 10^5^ cells/well and treating them with or without various stress inducers. Cells were lysed in RIPA buffer supplemented with a Protease and Phosphatase Inhibitor Cocktail and incubated on ice for 30 min. The lysates were then centrifuged at 13,000 rpm for 15 min at 4 °C, and the supernatants were collected. Protein concentration was determined using the Bradford assay^83^. A total of 40 µg of protein per sample was resolved by SDS-PAGE and transferred onto nitrocellulose membranes. Membranes were blocked for 1 h at room temperature in blocking buffer (5% w/v non-fat dry milk in TRIS-Buffered Saline pH 7.4 with 0.1% v/v Tween 20, TBST), then incubated overnight at 4 °C with specific primary antibodies diluted in blocking buffer. The antibodies used included anti-catalase, anti-PINK1, anti-DJ-1 and anti-β-actin (1:1000), anti-Parkin (2.5 µg/mL), anti-Nrf2 and anti-SOD2 (1:5000). After washing, membranes were incubated for 1 h at room temperature with the appropriate horseradish peroxidase (HRP)-conjugated secondary antibodies. Immunoreactive bands were visualized using a Chemidoc XRS detection system (Bio-Rad) and Image Lab software (4.0 version), with enhanced chemiluminescence substrates (ECL Star Enhanced Chemiluminescent Substrate or LiteAblot TURBO Extra Sensitive Chemiluminescent Substrate, EuroClone). Densitometric analysis of protein bands was performed using ImageJ software (1.50i version). Expression of β-actin was used as a loading control.

### Determination of mitochondrial membrane potential (ΔΨm)

Mitochondrial transmembrane potential (ΔΨm) was assessed by staining the cells with MitoTracker™ Red CMXRos dye (M7512, Thermo Fisher Scientific). pLV and pLV-URG7 cells were seeded in 24-well plates and treated as previously described. After 24 h of treatment, cells were harvested by trypsinization, washed with PBS and resuspended in fresh, prewarmed culture medium containing 300 nM MitoTracker dye. FCCP (5 µM) was used as positive control. Cells were incubated for 30 min at 37 °C, then washed again with PBS and analyzed by flow cytometry using a BD FACSCanto II system (BD Pharmingen, San Jose, CA, USA), with excitation at 485 nm and emission recorded between 579 and 599 nm. Unstained cells were used to establish baseline fluorescence, and forward/side scatter (FSC/SSC) parameters were applied to exclude cellular debris. A minimum of 10,000 events were recorded.

### Statistical analysis

Each experiment was performed at least in triplicate. Unless otherwise specified, results are expressed as the mean ± standard error of the mean (SEM). Statistical significance was determined using Student’s t-test or one-way ANOVA with appropriate corrections for multiple comparisons. A p-value of < 0.05 was considered statistically significant. Data analysis was conducted using GraphPad Prism software, version 8.4.2 (GraphPad; San Diego, CA).

## Supplementary Information

Below is the link to the electronic supplementary material.

Supplementary Material 1

## Data Availability

The datasets generated and/or analysed during the current study are available from the corresponding author on reasonable request. Original uncropped Western blot images are provided in the Supplementary Information.
