# Supplementary material for: Targeting oxidative stress and mitochondrial dysfunction via URG7 overexpression in an in vitro Parkinson’s disease neuronal model
Source: Sci Rep. 2026 Feb 19;16:9955. doi: 10.1038/s41598-026-38925-6 (PMC13022266; doi:10.1038/s41598-026-38925-6)
Supplement: Supplementary file 1 — Supplementary Material 1 [file 41598_2026_38925_MOESM1_ESM.pptx]

## Slide 1
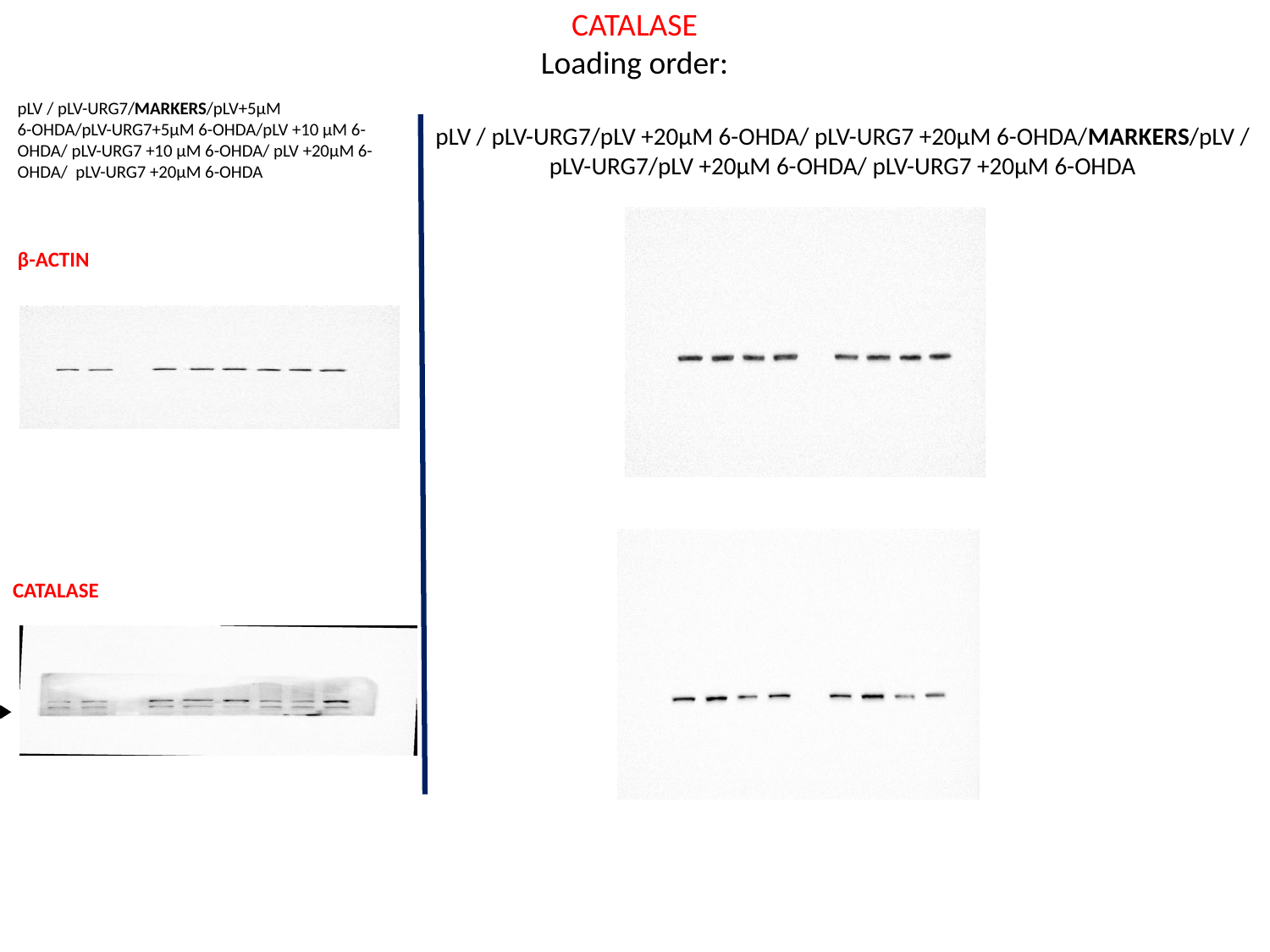

CATALASE
Loading order:
pLV / pLV-URG7/MARKERS/pLV+5µM 6-OHDA/pLV-URG7+5µM 6-OHDA/pLV +10 µM 6-OHDA/ pLV-URG7 +10 µM 6-OHDA/ pLV +20µM 6-OHDA/ pLV-URG7 +20µM 6-OHDA
pLV / pLV-URG7/pLV +20µM 6-OHDA/ pLV-URG7 +20µM 6-OHDA/MARKERS/pLV / pLV-URG7/pLV +20µM 6-OHDA/ pLV-URG7 +20µM 6-OHDA
β-ACTIN
CATALASE

## Slide 2
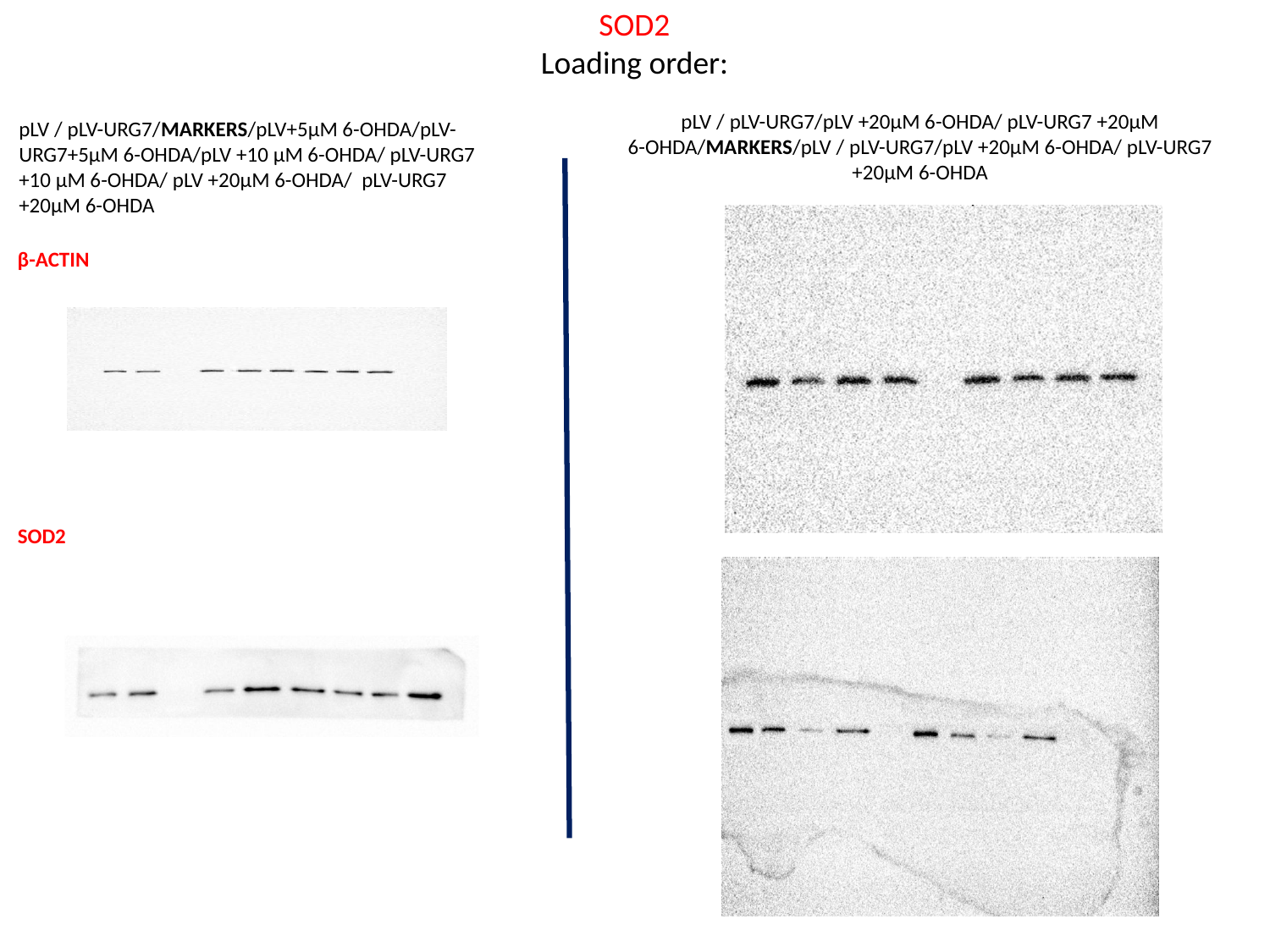

SOD2
Loading order:
pLV / pLV-URG7/pLV +20µM 6-OHDA/ pLV-URG7 +20µM 6-OHDA/MARKERS/pLV / pLV-URG7/pLV +20µM 6-OHDA/ pLV-URG7 +20µM 6-OHDA
pLV / pLV-URG7/MARKERS/pLV+5µM 6-OHDA/pLV-URG7+5µM 6-OHDA/pLV +10 µM 6-OHDA/ pLV-URG7 +10 µM 6-OHDA/ pLV +20µM 6-OHDA/ pLV-URG7 +20µM 6-OHDA
β-ACTIN
SOD2

## Slide 3
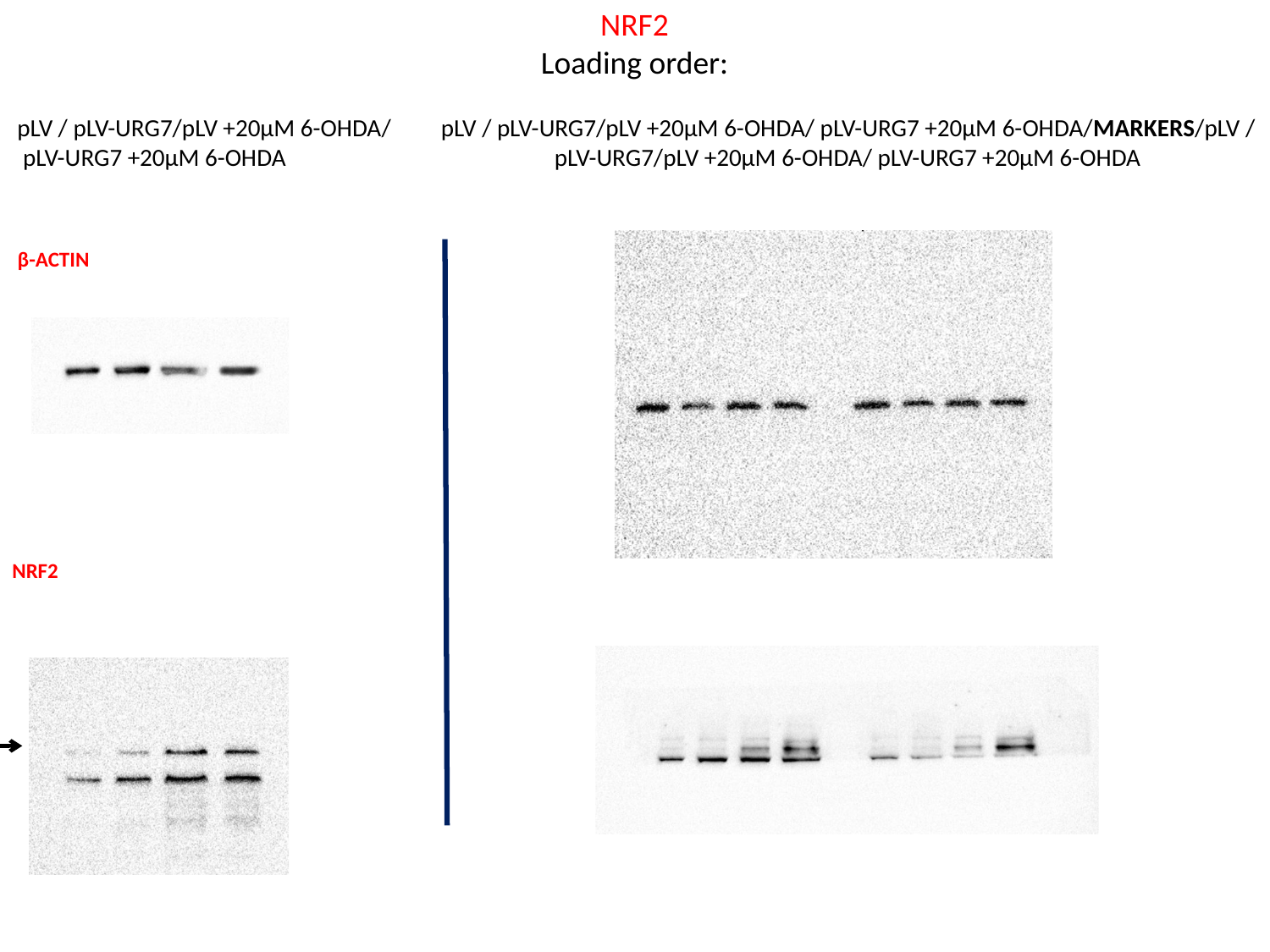

NRF2
Loading order:
pLV / pLV-URG7/pLV +20µM 6-OHDA/
 pLV-URG7 +20µM 6-OHDA
pLV / pLV-URG7/pLV +20µM 6-OHDA/ pLV-URG7 +20µM 6-OHDA/MARKERS/pLV / pLV-URG7/pLV +20µM 6-OHDA/ pLV-URG7 +20µM 6-OHDA
β-ACTIN
NRF2

## Slide 4
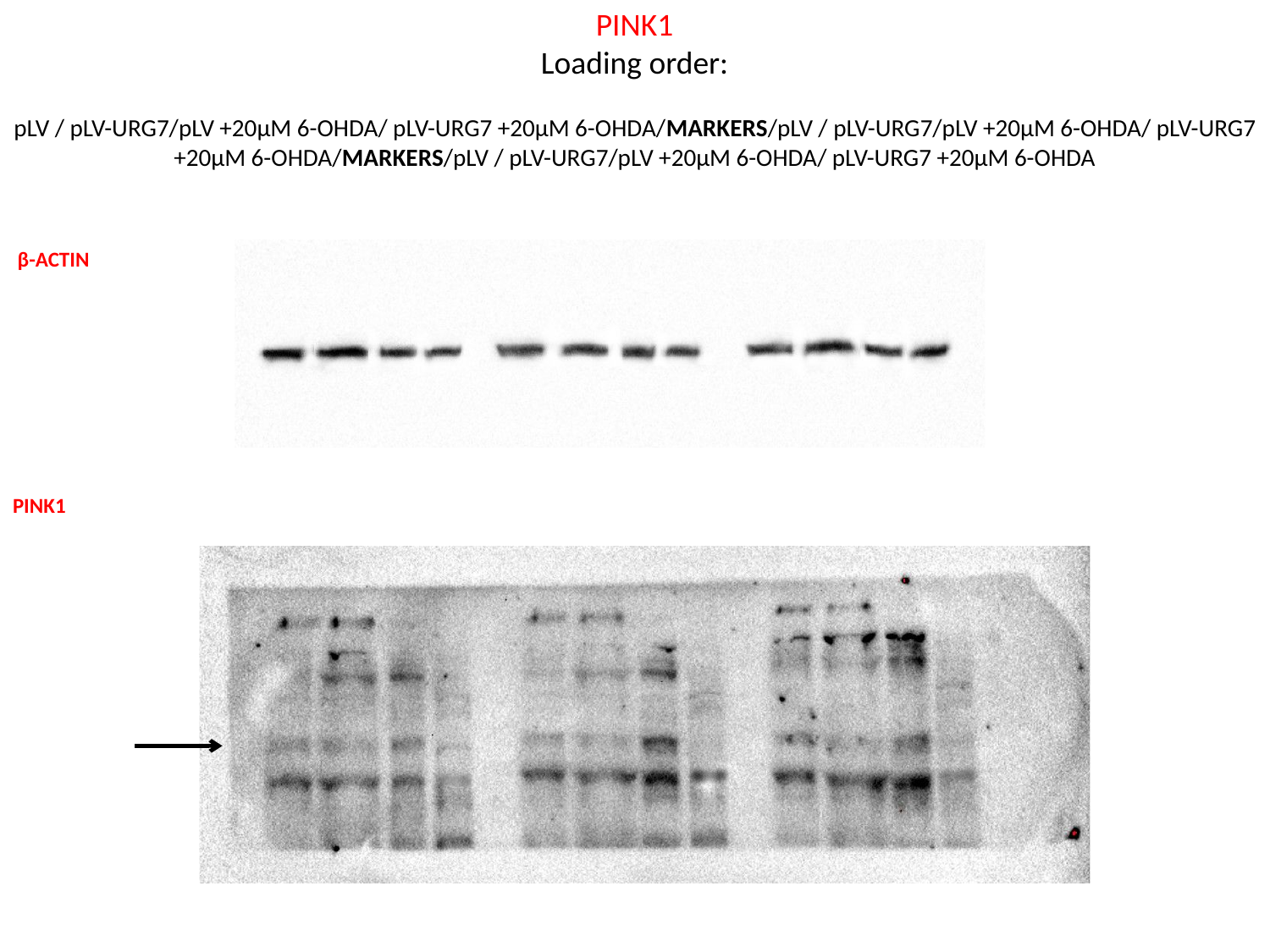

PINK1
Loading order:
pLV / pLV-URG7/pLV +20µM 6-OHDA/ pLV-URG7 +20µM 6-OHDA/MARKERS/pLV / pLV-URG7/pLV +20µM 6-OHDA/ pLV-URG7 +20µM 6-OHDA/MARKERS/pLV / pLV-URG7/pLV +20µM 6-OHDA/ pLV-URG7 +20µM 6-OHDA
β-ACTIN
PINK1

## Slide 5
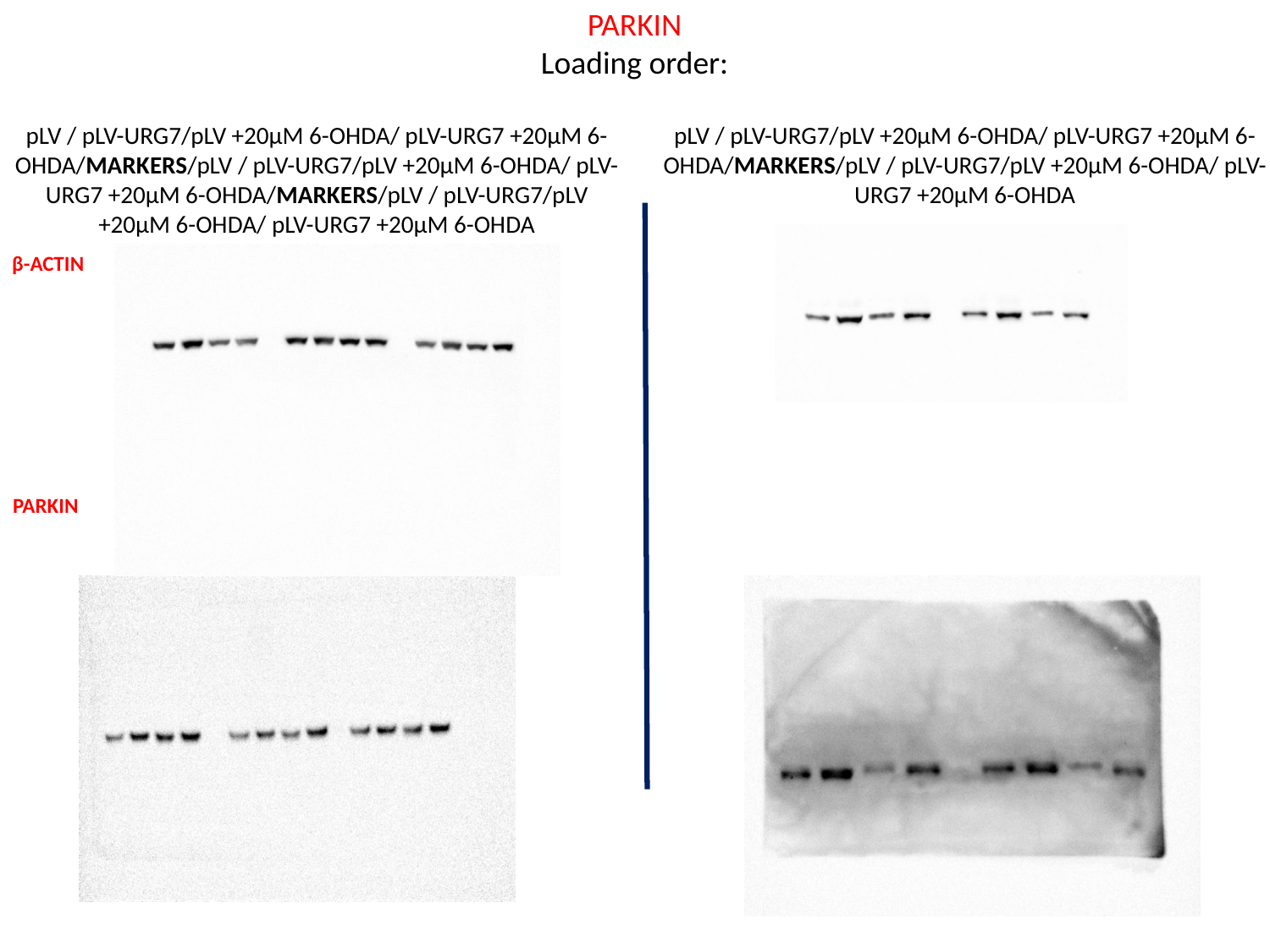

PARKIN
Loading order:
pLV / pLV-URG7/pLV +20µM 6-OHDA/ pLV-URG7 +20µM 6-OHDA/MARKERS/pLV / pLV-URG7/pLV +20µM 6-OHDA/ pLV-URG7 +20µM 6-OHDA/MARKERS/pLV / pLV-URG7/pLV +20µM 6-OHDA/ pLV-URG7 +20µM 6-OHDA
pLV / pLV-URG7/pLV +20µM 6-OHDA/ pLV-URG7 +20µM 6-OHDA/MARKERS/pLV / pLV-URG7/pLV +20µM 6-OHDA/ pLV-URG7 +20µM 6-OHDA
β-ACTIN
PARKIN

## Slide 6
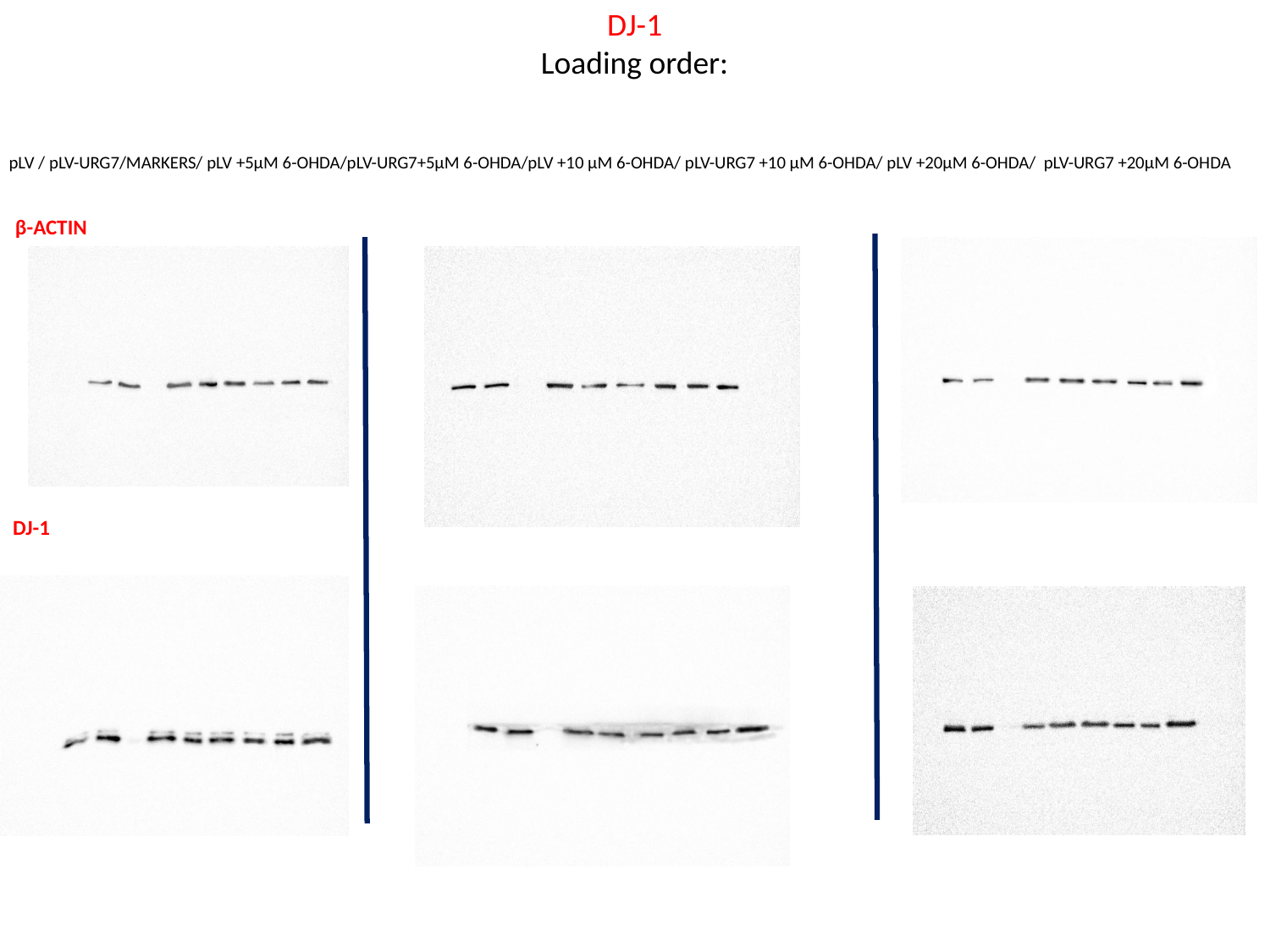

DJ-1
Loading order:
pLV / pLV-URG7/MARKERS/ pLV +5µM 6-OHDA/pLV-URG7+5µM 6-OHDA/pLV +10 µM 6-OHDA/ pLV-URG7 +10 µM 6-OHDA/ pLV +20µM 6-OHDA/ pLV-URG7 +20µM 6-OHDA
β-ACTIN
DJ-1
